# HIGH ON SMI: QUALITY CONCERNS ABOUT NURSING HOMES SERVING HIGH PROPORTIONS OF SERIOUSLY MENTALLY ILL RESIDENTS

**DOI:** 10.1093/geroni/igz038.1885

**Published:** 2019-11-08

**Authors:** Dylan J Jester, Kathryn Hyer, John R Bowblis

**Affiliations:** 1 School of Aging Studies, University of South Florida, Tampa, Florida, United States; 2 University of South Florida, Tampa, Florida, United States; 3 Department of Economics, Miami University, Oxford, Ohio, United States

## Abstract

The proportion of nursing home (NH) residents that have serious mental illness (SMI) has increased over the least two decades. Residents with SMI tend be younger and have different medical needs than traditional residents. To better understand this population, our study examined the facility, staffing, and resident characteristics of NHs that were more likely to specialize in SMI. Utilizing the Certification and Survey Provider Enhanced Reports, low-SMI (N = 3,616) and high-SMI (N = 3,615) NHs were defined as the first and fourth quartile of the distribution of the proportion of SMI residents, respectively. We performed bivariate tests and multivariate logistic regression to compare facility, staffing, resident, and star-ratings characteristics between NHs. High-SMI NHs were less likely to be Not-For-Profit, have fewer beds, have more Medicaid-paying residents, lower registered nurse staffing, and lower certified nurse aide staffing levels (p’s<.001). Residents in high-SMI NHs were more likely to require behavioral healthcare (p<.001) and be treated with psychoactive medications (any psychoactive, antidepressants, antipsychotics, anxiolytics (p’s<.001), hypnotics (p<.01)). Finally, high-SMI facilities had lower overall quality, health inspection, quality measure, staffing, and registered nurse staffing star-ratings (p’s<.001). High-SMI NHs have characteristics that are associated with lower quality-of-care (e.g., For-Profit, more Medicaid), lower staffing, prescribe more psychoactive medications, and have lower star-ratings. As the SMI population grows, large numbers of SMI residents will concentrate in a few NHs. While further research is needed to understand the implications of these trends, policy-makers must be aware of this population when affecting the resources and staffing of NHs.

